# Dermaseptin-PH: A Novel Peptide with Antimicrobial and Anticancer Activities from the Skin Secretion of the South American Orange-Legged Leaf Frog, *Pithecopus* (*Phyllomedusa*) *hypochondrialis*

**DOI:** 10.3390/molecules22101805

**Published:** 2017-10-24

**Authors:** Linyuan Huang, Dong Chen, Lei Wang, Chen Lin, Chengbang Ma, Xinping Xi, Tianbao Chen, Chris Shaw, Mei Zhou

**Affiliations:** 1 Natural Drug Discovery Group, School of Pharmacy, Queen’s University, Belfast BT9 7BL, Northern Ireland, UK; lhuang09@qub.ac.uk (L.H.); dchen03@qub.ac.uk (D.C.); l.wang@qub.ac.uk (L.W); clin011@163.com (C.L.); t.chen@qub.ac.uk (T.C.); chris.shaw@qub.ac.uk (C.S.); m.zhou@qub.ac.uk (M.Z.); 2 College of Basic Medical Science, Zhejiang Chinese Medial University, Hangzhou 310053, China

**Keywords:** dermaseptin, amphibian skin secretion, molecular cloning, antimicrobial, anticancer

## Abstract

The dermaseptin peptides, mainly derived from the skin secretions of Hylidae frogs, belong to a superfamily of antimicrobial peptides and exhibit diverse antimicrobial and anticancer activities with low cytotoxicity. Here, we reported a novel dermaseptin peptide, from the South American orange-legged leaf frogs, *Pithecopus* (*Phyllomedusa*) *hypochondrialis,* processing the shortest peptide length, namely Dermaseptin-PH. The complementary DNA (cDNA) encoding biosynthetic precursor of Dermaseptin-PH was initially identified by the rapid amplification of cDNA ends PCR (RACE-PCR) technique from the skin secretion. The predicted primary structure was confirmed by a combination of reverse-phase high performance liquid chromatography (RP-HPLC) and MS/MS fragmentation from the skin secretion. Chemically-synthetic Dermaseptin-PH was investigated using a range of bioactivity assessment assays to evaluate the biological activities and cytotoxicity of Dermaseptin-PH. Dermaseptin-PH inhibited the growth of Gram-negative bacteria, Gram-positive bacteria, and pathogenic yeast *Candida albicans*. In addition, Dermaseptin-PH showed a broad-spectrum of anticancer activities against several cancer cell lines including MCF-7, H157, U251MG, MDA-MB-435S, and PC-3. The potent antimicrobial and anticancer activities of Dermaseptin-PH make it a promising candidate in the discovery of new drugs for clinical applications, and the relatively short sequence of Dermaseptin-PH can provide new insight for the research and structural modification of new peptide drugs.

## 1. Introduction

Antimicrobial peptides (AMPs), as multifunctional host-defence peptides, have clear antibacterial functions against a series of bacteria, fungi, viruses, and parasites, and have gained widespread interest in recent years [1]. AMPs not only exist in the secretions of frogs and toads, but are also present in microorganisms, plants, animals, and even human bodies. In mammals, these short cationic peptides which are also called host-defence peptides (HDPs) act as natural antibiotics and participate in the immunoregulation or killing of pathogenic microorganisms after invasion [2,3]. Nevertheless, scientists found that HDPs are more inclined to induce the immune response mechanisms under physiological conditions to protect hosts from infection of pathogens, than to be directly involved in killing bacteria [4,5].

With the ever-increasing problems of antibiotic resistance and the emergence of superbacteria, the current clinical antibiotics cannot meet the demand of patients [6,7]. The various mechanisms of multidrug resistance limit the development of antibiotics. Some pathogens, including *Enterococcus faecium*, MRSA, and *Pseudomonas aeruginosa*, are drug resistant or multidrug resistant bacteria that threaten the life of immunocompromised sufferers. The ESKAPE pathogens which are comprised of six multi-drug resistant microbes are generally recommended for screening of antimicrobial drugs [8]. However, unlike the general mechanisms of most antibiotics targeting the particular enzymes in the process of pathogenic metabolism, AMPs display totally different antimicrobial mechanisms which make them one of the most promising anti-infective drug candidates that meet the customers’ need [9].

Besides, antimicrobial peptides with anticancer activities have been widely reported in some literatures, and apparently, as a result, are attracting scientists to investigate and discover new anticancer candidates. Although the development of anticancer peptides in clinical therapy is beset by difficulties, there are still a few AMPs undergoing clinical trials. LL-37 is a human source cathelicidin peptide which exists in human respiratory and reproductive system and skin [10]. A collaboration programme between the National Cancer Institute and the M.D. Anderson Cancer Centre, indicated that LL-37 was an efficient antitumour agent against melanoma, and was undergoing Phase II clinical trials at present [11]. Another candidate anticancer drug, LTX-315, an engineered lytic peptide, is currently in Phase I clinical trial for treatment against transdermally accessible tumours. It is worth noting that anticancer peptides can enhance therapeutic effects through combination with other therapeutic approaches, including surgery, chemotherapy, radiotherapy, and even immunotherapy. The combination of LTX-315 with immunotherapy for treatment of patients with transdermally accessible tumours is in the process of trials, which provide possibilities from more therapeutic aspects [12,13,14].

The overall objectives of this study were to isolate and identify a novel antimicrobial peptide, named Dermaseptin-PH, from the skin secretion of the South American orange-legged leaf frog, *Pithecopus* (*Phyllomedusa*) *hypochondrialis* [15], to measure the bioactivities of it and to assess its therapeutic potential. To this end, the complementary DNA (cDNA) encoding Dermaseptin-PH biosynthetic precursor was cloned by “shotgun” molecular cloning and subsequently, the predicted mature peptide was identified by reverse-phase high performance liquid chromatography (RP-HPLC) from the crude skin secretion and the primary structure was confirmed by MS/MS sequencing technique. After chemical synthesis of Dermaseptin-PH, antimicrobial activity was investigated using several Gram-negative and Gram-positive bacteria, and a pathogenic yeast included. In the meanwhile, anticancer activity was assessed on a wide range of cancer cell lines in vitro. The cytotoxicity of Dermaseptin-PH against eukaryotic cells was evaluated on horse red blood cells and human dermal microvascular endothelium cells.

## 2. Results

### 2.1. “Shotgun” Cloning of Dermaseptin-PH Precursor-Encoding cDNA from a Skin Secretion-Derived cDNA Library of Pithecopus hypochondrialis and Bioinformatic Analysis

**Figure 1:**
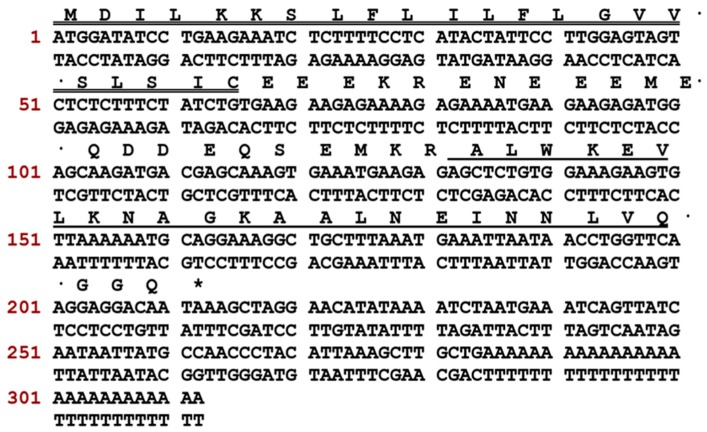
Nucleotide and translated open-reading frame amino acid sequences of Dermaseptin-PH from the skin secretion of *Pithecopus hypochondrialis*. The putative signal peptide is double-underlined, the mature peptide is single-underlined, and the stop codon is marked by an asterisk.

A full-length cDNA encoding one novel dermaseptin peptide named Dermaseptin-PH was identified from the cDNA library constructed by isolating mRNA from the skin secretion of *Pithecopus hypochondrialis.* The biosynthetic precursor of Dermaseptin-PH consisted of a hydrophobic signal peptide region followed by an acidic spacer and a single copy of the predicted mature peptide (Figure 1). The translated open-reading frame of this novel dermaseptin biosynthetic precursor consisted of 312 base pairs encode 70 amino acids. The prepropeptide comprises a 22-amino acid signal peptide region followed by three Glu residues, and a typical processing site Lys–Arg amino acid pairing. Following the remaining acidic spacer domain, another processing site Lys–Arg is showed before the mature Dermaseptin-PH, that contains 23 amino acid residues followed by an amidation consensus motif –Gly–Gly–Gln [16]. The nucleotide sequence of Dermaseptin-PH has been deposited in the GenBank Database (https://www.ncbi.nlm.nih.gov/genbank/) under the accession code MF805718.

**Figure 2:**
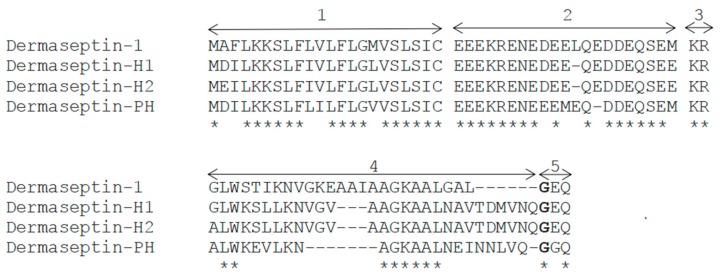
Alignments of the amino acid sequences of the prepropeptides of Dermaseptin-1, Dermaseptin-H1, Dermaseptin-H2, and Dermaseptin-PH comprise five domains, 1: putative N-terminal signal peptide region; 2: acidic amino acid rich spacer peptide region; 3: endoproteolytic cleavage point; 4: mature peptide progenitor region; 5: extension of biosynthetic precursor. Amino acid indicated with bold type serves as an amide donor for C-terminal amidation. The identical amino acid residues are indicated by the asterisks.

**Figure 3:**
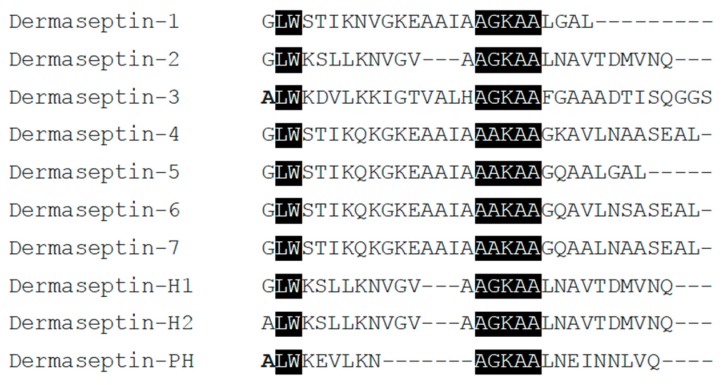
Alignments of amino acid sequences of Dermaseptin-1 (Accession No. P84596), Dermaseptin-2 (Accession No. P84597), Dermaseptin-3 (Accession No. P84598), Dermaseptin-4 (Accession No. P84599), Dermaseptin-5 (Accession No. P84600), Dermaseptin-6 (Accession No. P84601), Dermaseptin-7 (Accession No. P84880), Dermaseptin-H1 (Accession No. Q0VZ36), Dermaseptin-H2 (Accession No. Q0VZ37) and Dermaseptin-PH derived from the frog *Pithecopus hypochondrialis* are displayed. The identical amino acids are shaded in black; the amino acid with slight changes are indicated in bold type; the gaps are used to maximise alignments.

Additionally, the alignments of the full length prepropeptides of Dermaseptin-PH with Dermaseptin-1, Dermaseptin-H1, and Dermaseptin-H2 were displayed below (Figure 2). The sequences showed a highly conserved signal peptide domain consisting of 22 amino acid residues and the acidic spacer region comprising 20–21 residues. Besides, they have a –GGQ or –GEQ extension at C-terminal. The mature peptide amino acid sequences derived from the same species were aligned in Figure 3. An identical Trp residue in the position three at N-terminal and a consensus motif of –AA/GKAA– amphipathic structure in the middle region was observed.

### 2.2. Identification and Structural Characterization of Dermaseptin-PH in Skin Secretion of Pithecopus hypochondrialis

**Figure 4:**
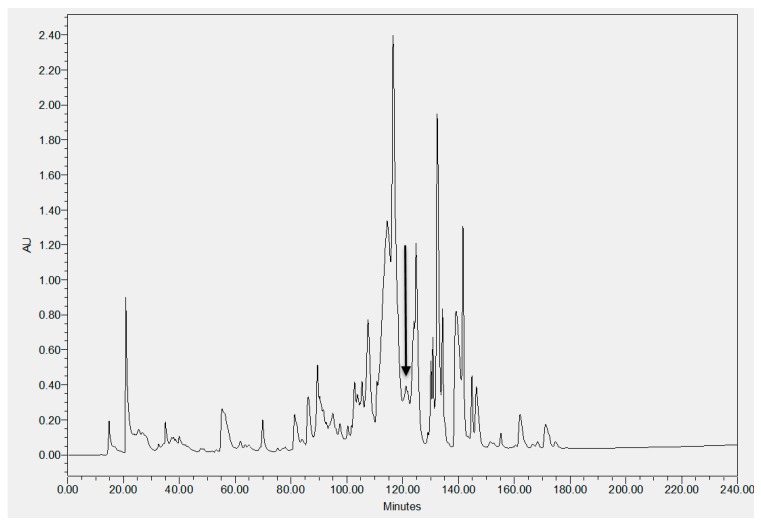
Reverse-phase high performance liquid chromatography (RP-HPLC) chromatogram of lyophilised skin secretion of *Pithecopus hypochondrialis*. The arrow indicates the retention time/elution position of the fraction containing a peptide with a mass coincident with that of Dermaseptin-PH. The absorbance was set at *λ* = 214 nm. The *X*-axis indicates the retention time in minutes and the *Y*-axis indicates the relevant absorbance in arbitrary units.

**Figure 5:**
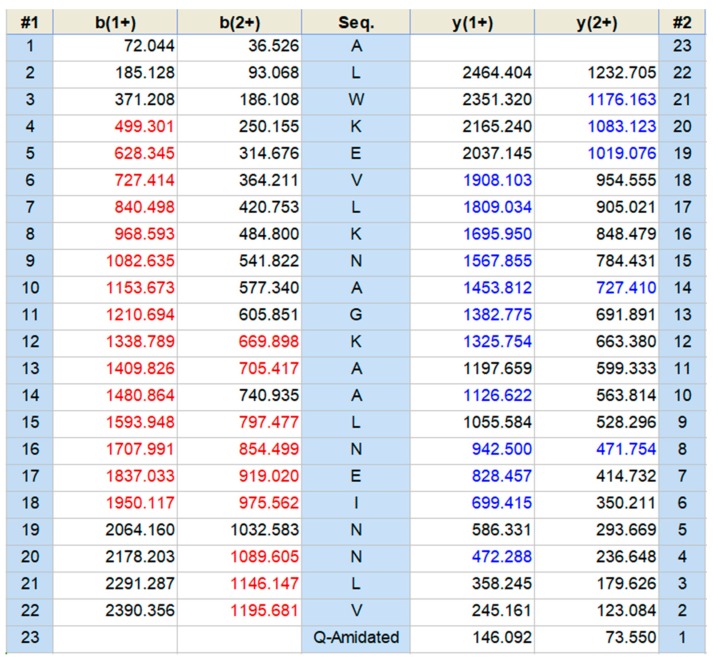
MS/MS fragmentation datasets derived from ions coincident with the mass of Dermaseptin-PH. Predicted singly and doubly charged *b*-ions and *y*-ions arising from MS/MS fragmentation of Dermaseptin-PH. Ions detected by MS/MS fragmentation are indicated in red and blue.

The fractions from the lyophilised crude skin secretion of *Pithecopus hypochondrialis* were eluted by RP-HPLC and fractions containing different natural components were collected every minute (Figure 4). Each fraction was analysed by matrix-assisted laser desorption ionization time-of-flight mass spectrometer (MALDI-TOF-MS), and the fraction with a mass coincident with that of the predicted mature peptide identified by the genomic study, is indicated by an arrow. The amino acid sequence of mature peptide, Dermaseptin-PH, was further confirmed by MS/MS fragmentation sequencing (Figure 5). The amidated C-terminal post-translational modification with the Gly residue following the C-terminal amino acid residue of the mature peptide domain was also verified in the sequencing result.

### 2.3. Circular Dichroism Spectroscopy

**Figure 6:**
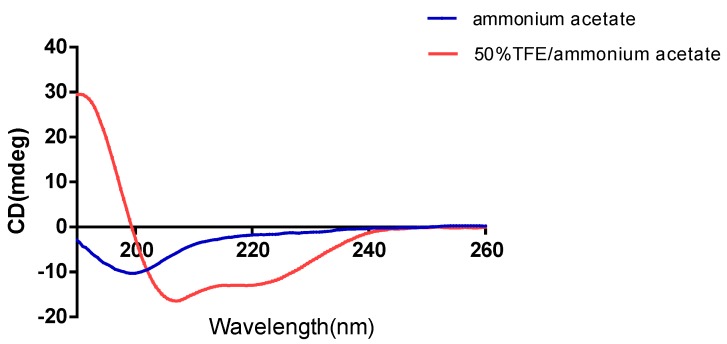
Circular dichroism (CD) spectra of Dermaseptin-PH (50 μM) in 10 mM ammonium acetate/water solution (blue line) and in 50% 2,2,2-trifluoroethanol (TFE)/10 mM ammonium acetate/water solution (red line).

The secondary structure of Dermaseptin-PH was successfully examined by circular dichroism spectroscopy in both aqueous and membrane-mimic environment (Figure 6). Dermaseptin-PH showed a random coil conformation in ammonium acetate/water solution, while it exhibited a typical α-helical structure in 50% 2,2,2-trifluoroethanol (TFE)/10 mM ammonium acetate/water solution. The helicity of Dermaseptin-PH was calculated as 35% by DichroWeb webserver [17].

### 2.4. Antimicrobial Activity and Haemolytic Activity of Dermaseptin-PH

**Figure 7:**
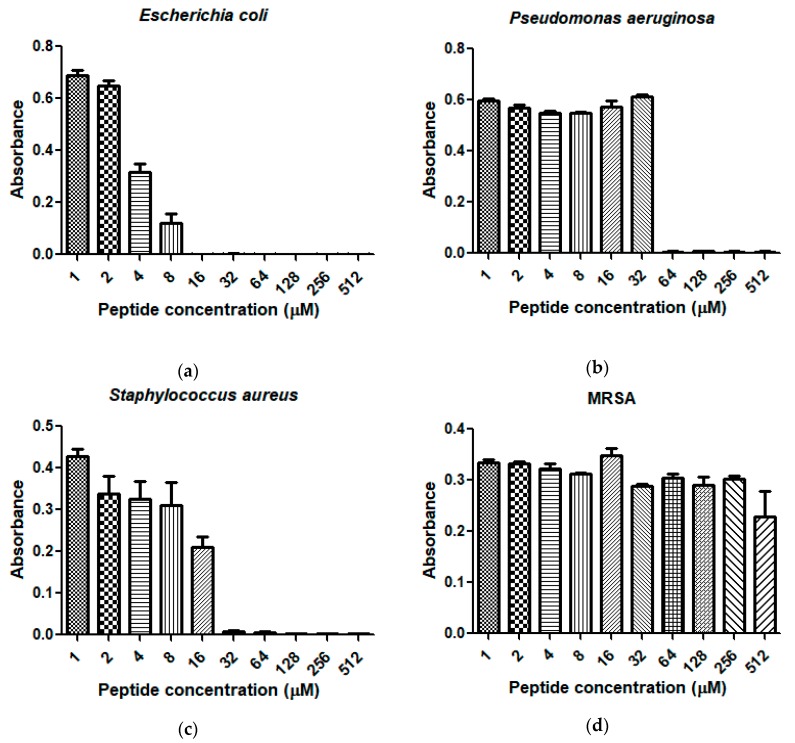

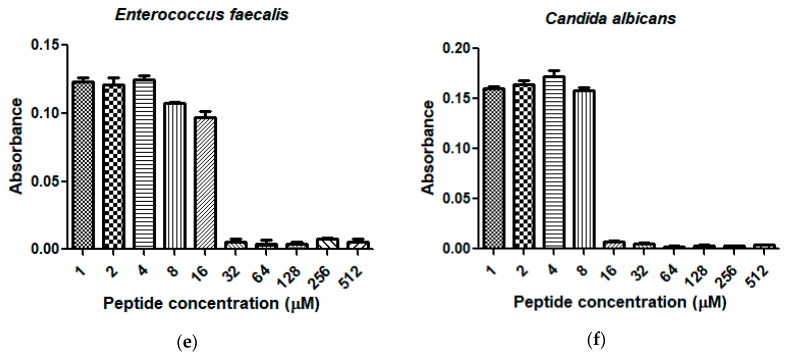
The antimicrobial activities of Dermaseptin-PH against the growth of (**a**) *E. coli*; (**b**) *P. aeruginosa*; (**c**) *S. aureus*; (**d**) MRSA; (**e**) *E. faecalis*; and (**f**) *C. albicans* in a range of concentrations from 1 μM to 512 μM. Data represent means ± stand error of the mean (SEM) of 5 replicates.

**Figure 8:**
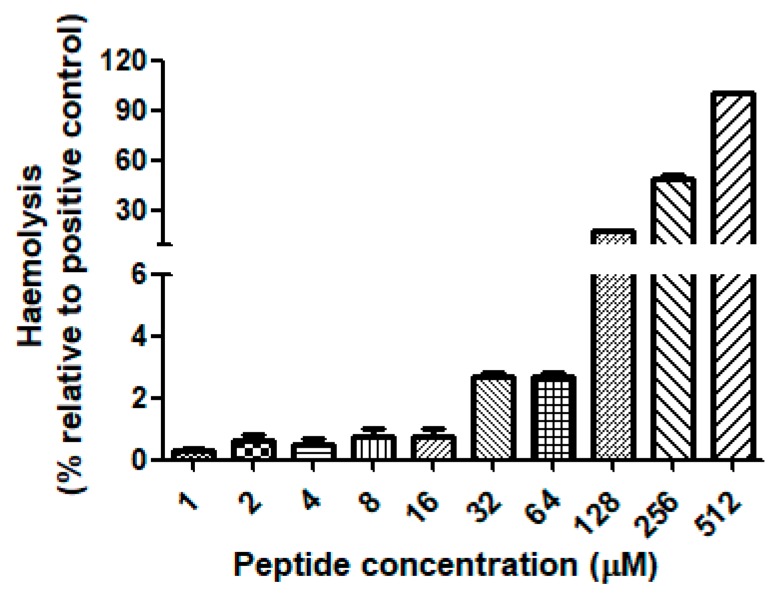
The haemolytic activity of Dermaseptin-PH was tested on horse red blood cells. The positive control group was treated with 1% Triton X-100 lysis buffer.

Dermaseptin-PH was demonstrated to have a broad-spectrum of antimicrobial activity against the Gram-negative bacterium, *Escherichia coli*; the Gram-positive bacterium, *Staphylococcus aureus*; and the pathogenic yeast, *Candida albicans*, with minimum inhibitory concentration (MIC) results ranging from 16 to 32 μM and minimum bactericidal concentration (MBC) results ranging from 16 to 64 μM (Table 1). The antimicrobial activity of Dermaseptin-PH against the tested microorganisms was showed in Figure 7. Dermaseptin-PH showed more effective potency to *E. coli* and *C. albicans* at the concentration of 16 μM, than to *S. aureus* with 32 μM. It is also important to note that Dermaseptin-PH exhibited more effective antimicrobial activity against *E. coli* (16 μM) than ampicillin (Amp) (36.6 μM). However, Dermaseptin-PH displayed lower efficiency than norfloxacin (Nor) on both MIC and MBC results against the six tested microorganisms. In addition, it is worthy of note that Dermaseptin-PH showed low cytotoxicity to mammalian red blood cells at its effective concentration against microorganisms in a range of concentrations from 16 to 64 μM (Figure 8).

### 2.5. Anti-Biofilm Activity of Dermaseptin-PH

**Figure 9:**
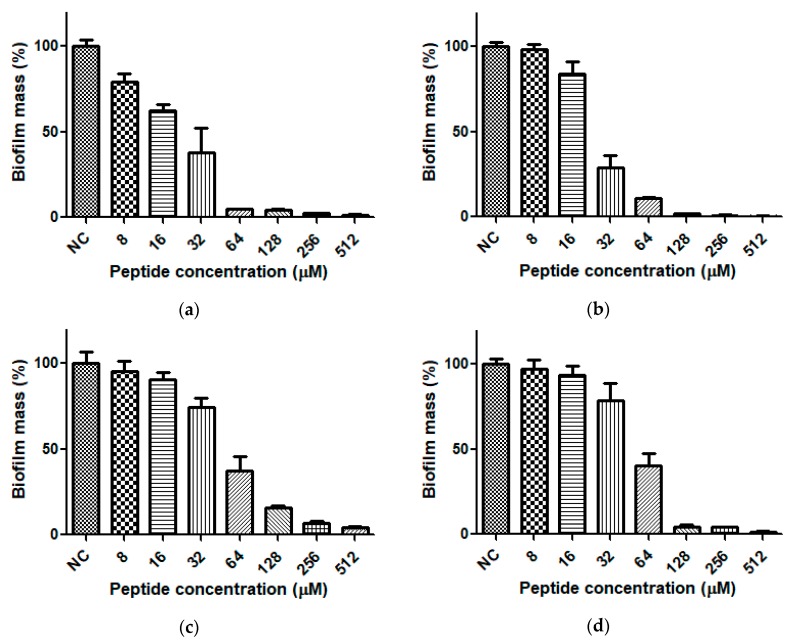
The biofilm formation inhibitory activity of Dermaseptin-PH against (**a**) *E. coli* and (**b**) *S. aureus*, and the biofilm eradication activity of Dermaseptin-PH against (**c**) *E. coli* and (**d**) *S. aureus*. Negative control was obtained following incubation with relevant culture medium. Data represent means ± SEM of 5 replicates.

Dermaseptin-PH exhibited moderate antibiofilm activities against both *E. coli* and *S. aureus* (Figure 9). It processed the same potency on the inhibition of biofilm of both strains, but showed 2-fold more effective activity when eradicating 90% mature biofilm of *S. aureus* (Table 2).

### 2.6. Bacteria Cell Membrane Permeabilisation Activities of Dermaseptin-PH

**Figure 10:**
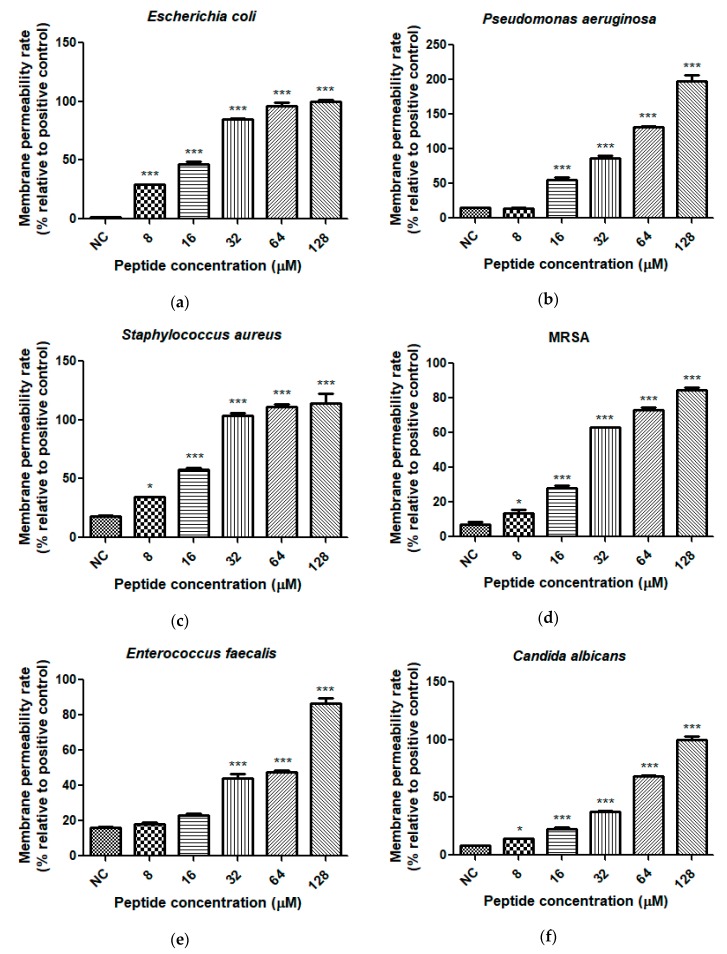
Cell membrane permeability effects of Dermaseptin-PH on (**a**) *E. coli*; (**b**) *P. aeruginosa*; (**c**) *S. aureus*; (**d**) MRSA; (**e**) *E. faecalis*; and (**f**) *C. albicans*, by using SYTOX Green (Life technologies, Carlsbad, CA, USA) assay at peptide concentrations corresponding to MIC results. Positive control was obtained following incubation with 70% isopropyl alcohol. Negative control (NC) was obtained following incubation with 5% tryptic soy broth (TSB) in 0.85% NaCl solution. Data represent means ± SEM of 5 replicates. The levels of significance are: * *p* < 0.05, *** *p* < 0.001.

The cell membrane permeability of Dermaseptin-PH was tested in a range of concentrations from 8 μM to 128 μM (Figure 10). Dermaseptin-PH showed more effective permeable activity on *E. coli*, *S. aureus*, MRSA, and *C. albicans* at concentration of 8 μM. The efficient permeabilisation concentrations of Dermaseptin-PH on *P. aeruginosa* and *E. faecalis* were two times and four times higher, at 16 and 32 μM, respectively. At the MICs against the respective microorganisms, the permeability rate of Dermaseptin-PH on *E. coli*, *P. aeruginosa*, *S. aureus*, *E. faecalis*, and *C. albicans* was 46.4%, 131.5%, 103.6%, 44.3%, and 22.9%. And, the permeability rate of Dermaseptin-PH on *E. coli*, *S. aureus*, *E. faecalis*, and *C. albicans* was 46.4%, 110.8%, 47.8%, and 37.4% at the respective MBCs. Each assay was carried out over at least three individual experiments, with 5 replicates in each.

### 2.7. Cell Proliferation Assay of Dermaseptin-PH

**Figure 11:**
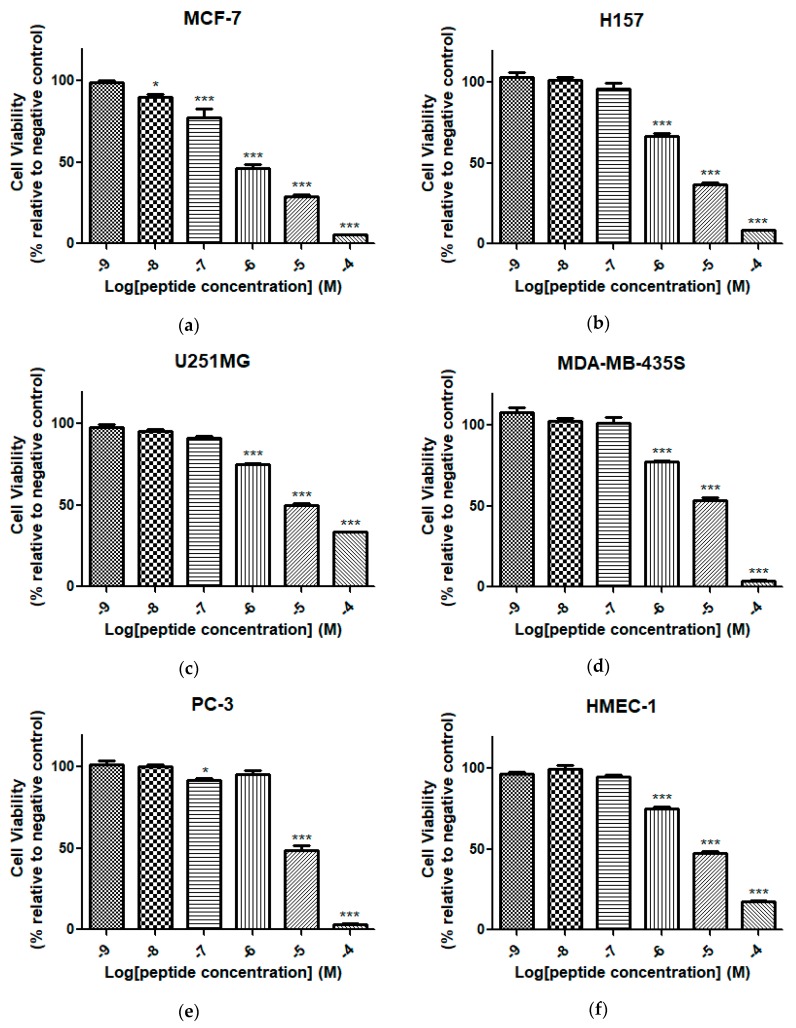
The effects of Dermaseptin-PH on proliferation of human cancer cell lines (**a**) MCF-7; (**b**) H157; (**c**) U251MG; (**d**) MDA-MB-435S; and (**e**) PC-3 and (**f**) human dermal microvascular endothelium cell HMEC-1; after treatment with Dermaseptin-PH for 24 h at a range of concentrations from 10^−9^ to 10^−4^ M. The levels of significance are: * *p* < 0.05, *** *p* < 0.001. The negative control group was treated with relevant serum-free culture medium.

Synthetic peptide Dermaseptin-PH exhibited a broad-spectrum antiproliferative activity against MCF-7, H157, U251MG, MDA-MB-435S, and PC-3 human cell lines in a range of concentrations from 10^−9^ to 10^−4^ M (Figure 11). The most obvious proliferation inhibitions observed were on the human cancer cell lines MCF-7 with IC_50_ value of 0.69 μM. Besides, Dermaseptin-PH showed similar inhibitory activities against H157, U251MG, and MDA-MB-435S cell lines, with IC_50_ values of 2.01, 2.36, and 9.94 μM, respectively, and weak anticancer activity against PC-3 cell lines, with an IC_50_ value of 11.8 μM. In addition, it is worth noting that Dermaseptin-PH showed slight cytotoxicity on human dermal microvascular endothelium cells, HMEC-1, with an IC_50_ value of 4.85 μM.

### 2.8. Cancer Cell Membrane Permeabilisation Activities of Dermaseptin-PH

**Figure 12:**
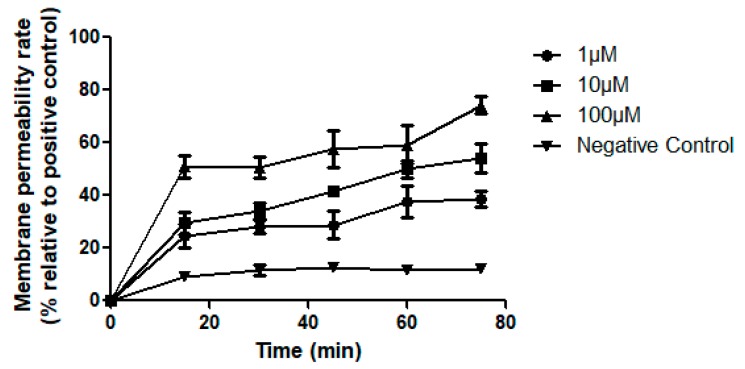
Cell membrane permeability effects of Dermaseptin-PH on MCF-7 cell by using SYTOX Green (1 μM) (Life technologies) at peptide concentrations from 1 to 100 μM. Positive control was obtained following incubation with 0.5% Triton X-100. Negative control was obtained following incubation with relevant serum-free culture medium. Data represent means ± SEM of 5 replicates.

The cell membrane permeability of Dermaseptin-PH was tested in a range of concentrations after incubation with MCF-7 cells for 75 min (Figure 12). The fluorescence detected at initial stage demonstrated an immediate damage on cancer cell membrane the moment that Dermaseptin-PH was added. Each assay was carried out over at least three individual experiments, with 5 replicates in each.

## 3. Discussion

Dermaseptins, which belong to an important antimicrobial peptide family, have a wide spectrum of antimicrobial activity against various microorganisms, including bacteria, fungi, enveloped viruses, protozoa, and mollicutes [18]. The first dermaseptin peptide named Dermaseptin S1 was discovered by Mor from frog, *Phyllomedusa sauvagii* [19]. This novel 34 amino acid residue peptide displayed lethal activity against pathogenic microorganisms, including filamentous fungi, which is the main reason for opportunistic infections of immunocompromised patients. In this study, we isolated a novel dermaseptin peptide, namely Dermaseptin-PH, from the skin secretion of the South American orange-legged leaf frog, *Pithecopus hypochondrialis*. Compared to the alignments of the amino acid sequences of the preprodermaseptins, Dermaseptin-PH shows similar structural characteristics to other members with a conserved signal peptide region, which constitutes 22 amino acids as canonical architecture. However, the variations of signal peptide region of dermaseptin peptide from same species were observed [20,21,22], which might be explained by the mutation occurring due to the different distribution of specimen. Additionally, the canonical structure in the mature peptide comprises an identical Trp residue in position three and a conserved –AA(A/G)KAAL(G/N)A– amphipathic domain. Besides, the C-terminal amide could influence the activities of AMPs because of 1+ net positive charge of amide group, and resistance to natural degradation [23]. Moreover, a Val–Gly amino acid pairing exists in the middle region of Dermaseptin B2, which can divide the amphipathic helix into N-terminal domain and C-terminal domain, and this may promote the penetration into the membrane of bacteria [24]. However, the Val–Gly amino acid pairing is absent in Dermaseptin-PH, which may decrease the antimicrobial activity.

AMPs derived from amphibian skins, as the initial defence protecting themselves from invasion of pathogens, have a potent and broad-spectrum antimicrobial activity. These low molecular weight peptides are regarded as a potential source for developing new antibiotics to solve the drug resistance caused by conventional antibiotics [25]. Dermaseptin-PH demonstrated a broad-spectrum antimicrobial activity against Gram-negative, Gram-positive bacteria, and yeast. The antimicrobial mechanism of dermaseptins has been reported to bind to lipid bilayers by coil-to-helix transition, and induce the permeation/disruption of the lipid plasma membrane through the carpet model [18]. It could explain the weaker antimicrobial potency of Dermaseptin-PH than Dermaseptin S9 as Dermaseptin S9 has a larger proportion of the *α*-helical domain than Dermaseptin-PH [26]. Dermaseptin-PH displayed more effective permeabilisation activity on *E. coli* than on *P. aeruginosa* may also owe to the more resistant cellular envelop structure of *P. aeruginosa*. Unexpectedly, Dermaseptin-PH exhibited permeation on MRSA though it was not able to inhibit growth of MRSA in a suitable growing environment. It is speculated that Dermaseptin-PH form the transmembrane pores rapidly, but this crisis in the membrane might be relieved by some kind of “self-fixing” strategy or enzymatic degradation of peptides. This special phenomenon might be due to that incident mutations of MRSA promote the adaption of harsh environment [27]. On the other hand, the acting pattern of Dermaseptin-PH on MRSA indicated that it may permeabilise cell membranes through other pore formation models instead of the carpet model. Although Dermaseptin-PH can interact with cell membranes and cause permeabilisation, its antimicrobial activity was significantly decreased when encountering more complex bacteria strains and propagation circumstances, such as biofilm-sessile bacteria. It is widely accepted that high cationic charge may play a more important role in antimicrobial activity of the most antimicrobial peptides. The published literature revealed that the most common approach to enhance antimicrobial activity is to introduce basic amino acids in the amino acids sequence, like lysine. Whereas antimicrobial results of Dermaseptin-PH may illustrate it exerts antimicrobial functions more dependent on amphipathicity, than positively charged features, upon interaction with the cell membrane.

Many AMPs display anticancer activity so that they attract much attention of researchers on anticancer drug discovery [28]. The mechanisms of antimicrobial peptides exhibiting anticancer activity such as disrupting cell membranes, inhibiting the growth of cancer cells, or killing cancer cells, have not yet been studied thoroughly. Some literature declared that some AMPs can kill tumour cells specifically. One reason is that the negatively-charged substances, like heparin sulphates and *O*-glycosylated mucins on the surface of cancer cells, could favour the interaction between AMPs and tumour cells [29,30,31,32,33]. Meanwhile, some scientists deduced that electrostatic force is not the decisive factor of the interaction between AMPs and cancer cells [34,35]. Although studies showed that some AMPs can induce cell apoptosis on eukaryote by reactive oxygen species (ROS) production, caspase activation, cytochrome *c* release, DNA fragmentation, and mitochondrial membrane depolarisation [36,37], Dermaseptin B2 was reported to induce the cell necrosis by the membrane disruption of cancer cells [38]. Here, considering Dermaseptin-PH contains less cationic charges and low degree of *α*-helicity, a further study of the permeabilisation of Dermaseptin-PH on cancer cells was carried out through a time course assay. By observation of rapid membrane permeabilisation activity on cancer cells, it was speculated that Dermaseptin-PH possesses similar permeation/disruption mechanisms for both cancer and bacteria cells. Interestingly, the interaction between Dermaseptin-PH and cancer cell membrane entered a slow growth period, after the initial rapid rise duration. This phenomenon may be explained by the long-lasting light irradiation from the light source inside the plate reader [39], and the lack of appropriate growth conditions. It might cause cell cytological instability, which makes cancer cells more sensitive to Dermaseptin-PH.

In summary, we reported the identification and characterisation of a novel dermaseptin peptide, named Dermaseptin-PH, from the skin secretion of the South America orange-legged leaf frog, *Pithecopus hypochondrialis*. Dermaseptin-PH showed a broad-spectrum of antimicrobial and anticancer activities. The cell membrane permeability against bacteria and cancer cells indicated that Dermaseptin-PH stands a good chance of a permeation/disruption dependent peptide. However, concerning the diverse structures of dermaseptins, the bioactivities may also be relevant to their structures. Hence, whether functions of dermaseptins have a strong connection with their structures still need to be investigated intensively. The discovery of AMPs with multiple functions brought a new possibility for the treatment of human disease. Although there are shortcomings for the clinical application of AMPs, there is still a strong confidence for the prospect of their therapeutic applications.

## 4. Materials and Methods

### 4.1. Acquisition of Pithecopus hypochondrialis Skin Secretion

Specimens of the South American orange-legged leaf frog, *Pithecopus hypochondrialis* were obtained as captive-bred adults, and were settled into their new surroundings for least three months prior to experimentation. All specimens were housed separately in purpose designed terraria under 12 h/12 h light/dark cycles, and were fed multivitamin-loaded crickets three times per week. Skin secretions were obtained by transdermal electrical stimulation after the method of Tyler et al. and these were washed from the skin using de-ionised water, snap-frozen in liquid nitrogen, lyophilised, and stored at 20 °C prior to analysis [40]. The study was performed according to the guidelines in the UK Animal (Scientific Procedures) Act 1986, project license PPL 2694, issued by the Department of Health, Social Services and Public Safety, Northern Ireland. Procedures had been vetted by the IACUC of Queen’s University Belfast, and approved on 1 March 2011.

### 4.2. “Shotgun” Cloning of Dermaseptin-PH Precursor-Encoding cDNA from a Skin Secretion-Derived cDNA Library of Pithecopus hypochondrialis

Five milligrams of lyophilised skin secretion were dissolved in 1 mL lysis/binding buffer (Dynal Biotech, Merseyside, UK). Polyadenylated mRNA was isolated by using a magnetized Dynabeads^®^ mRNA Direct^TM^ Kit (Dynal Biotech, Merseyside, UK) from the decanted supernatant. Subsequently, the first-strand cDNA was synthesised, and the rapid amplification of cDNA ends PCR (RACE-PCR) was performed to obtain the full length of prepropeptide nucleic acid sequence using a SMART-RACE Kit (Clontech, Palo Alto, CA, USA). The nested universal primer supplied with the kit was used by combination of the degenerate sense primer (S1; 5′-ACTTTCYGAWTTRYAAGMCCAAABATG-3′) (Y = C/T; W = A/T; R = A/G; M = A/C; B = T/C/G) that was designed based on the highly conserved signal peptide regions of dermaseptin peptides published previously from subfamily Phyllomedusinae. Purification of PCR products were carried out using a Concert^TM^ Rapid PCR Purification System (Life Technologies, Warrington, UK) and the products were cloned using a pGEM^®^-T Easy Vector system (Promega Corporation, Southampton, UK). And an ABI 3100 automated capillary sequencer (Applied Biosystems, Foster City, CA, USA) was used to analyse the nucleotide sequence of selected cloned products.

### 4.3. Identification and Structural Characterization of the Putative Dermaseptin-PH from the Skin Secretion of Pithecopus hypochondrialis

Five mg of lyophilised skin secretion from *Pithecopus hypochondrialis* were dissolved in 1 mL of trifluoracetic acid (TFA)/water (0.05/99.95, *v*/*v*) and centrifuged at 5000× *g* for 20 min to clarify. The supernatant was injected into an RP-HPLC system (Waters, Miford, MA, USA) fitted with a column (Jupiter C-5, 5 μM, 4.6 mm × 250 mm, Phenomenex, Macclesfield, Cheshire, UK) and eluted with a linear gradient mobile phase from TFA/waster (0.05/99.95, *v*/*v*) to TFA/water/acetonitrile (0.05/19.95/80.00, *v*/*v*/*v*) at a flow rate of 1 mL/min in 240 min, detected by the ultraviolet absorbance at 214 nm. Each fraction was collected automatically at 1 min intervals, and then analysed by a MALDI-TOF-MS (Voyager DE, Perspective Biosystems, Foster City, CA, USA) in positive detection mode, using *α*-cyano-4-hydroxycinnamic acid (CHCA) as the matrix. The system was calibrated by standards with precision reaching ± 0.1%. Fractions containing identical molecular masses correspond with the deduced mature peptide from the cloned cDNA were injected into to an LCQ-Fleet electrospray ion-trap mass spectrometer (Thermo Fisher Scientific, San Francisco, CA, USA) to analysis sequence structure by MS/MS fragmentation sequencing technique.

### 4.4. Solid-Phase Peptide Synthesis

Peptide was chemically-synthesised by solid-phase Fmoc Chemistry using a Tribute^®^ PS4 automated solid-phase peptide synthesizer (Protein Technologies, Tucson, AZ, USA) for bioactive assessment after the primary structure of the peptide was confirmed. Purity result of the synthesised peptide was identified by RP-HPLC and MALDI-TOF-MS reaching up to 95%.

### 4.5. Determination of Dermaseptin-PH Second Structure Using Circular Dichroism Spectroscopy

The secondary structure of Dermaseptin-PH was determined using a JASCO J-815 CD spectrometer (Jasco, Essex, UK). Chemically-synthesised peptide was dissolved in 10 mM ammonium acetate and 10 mM ammonium acetate with 50% TFE, respectively, and was prepared at 50 μM in a 1 mm precision quartz cell (Hellma Analytics, Essex, UK). CD spectra were recorded at a wavelength ranging from 190 nm to 260 nm with a 200 nm/min scan speed. The parameters were set as 1 nm bandwidth and 0.5 nm data pitch and the results were analysed by the DichroWeb webserver to estimate *α*-helical content [17].

### 4.6. Antimicrobial Assays

Antimicrobial activity of synthetic Dermaseptin-PH was assessed by determination of MIC and MBC assays using six standard bacteria, including Gram-negative bacteria *Escherichia coli* (NCTC 10418), and *Pseudomonas aeruginosa* (ATCC 27853); Gram-positive bacteria *Staphylococcus aureus* (NCTC 10788), methicillin-resistant *Staphylococcus aureus* (MRSA) (NCTC 12493), and *Enterococcus faecalis* (NCTC 12697); and pathogenic yeast *Candida albicans* (NCYC 1467). Each microorganism was inoculated in Mueller–Hinton broth (MHB) (Sigma-Aldrich, St. Louis, MO, USA) for 16–18 h, and then subcultured until reaching the logarithmic growth phase, by measuring optical density (OD) of the cultures at wavelength 550 nm. Then, the cultures were diluted to obtain 1 × 10^6^ colony forming units (cfu)/mL for the bacteria, and 1 × 10^5^ cfu/mL for the yeast, and incubated with various concentrations of Dermaseptin-PH, with final concentration range from 1 to 512 μM in a 96 well microtiter cell culture plate. Plates were incubated for 18 h at 37 °C in a humid atmosphere, and the growth of bacteria/yeast was determined by means of measuring absorbance values at wavelength 550 nm using a Synergy HT plate reader (Biotech, Minneapolis, MN, USA). The MIC results were defined as the lowest concentrations of peptide at which no distinct growth of the microorganisms was detectable. For the MBC assay, the culture in each well was spotted onto a Mueller–Hinton agar (MHA) (Sigma-Aldrich, St. Louis, MO, USA) plate, and incubated at 37 °C in a humid atmosphere for 18 h. The MBC results were defined as the lowest concentrations of peptide at which no obvious colony was observed.

### 4.7. Minimum Biofilm Inhibitory Concentration (MBIC) and Minimum Biofilm Eradication Concentration (MBEC) Assays

Gram-negative bacterium *Escherichia coli* (NCTC 10418) was inoculated in Luria–Bertani (LB) broth (Sigma-Aldrich, St. Louis, MO, USA); Gram-positive bacteria *Staphylococcus aureus* (NCTC 10788) was inoculated in tryptic soy broth (TSB) (Sigma-Aldrich, St. Louis, MO, USA) for 16–18 h, and then subcultured until reaching the logarithmic growth phase by measuring OD of the cultures at wavelength 550 nm. For the MBIC assay, the cultures were diluted to obtain 1 × 10^6^ cfu/mL, and incubated with various concentrations of Dermaseptin-PH with final concentration range from 1 to 512 μM in a 96 well flat-bottomed microtiter cell culture plate for 16 to 20 h at 37 °C in a shaking incubator at different revolutions per minute (rpm), according to different bacteria. After, plates were washed twice by phosphate buffered saline (PBS) and fixed with methanol for 10 min. For the MBEC assay, 100 μL of the cultures were placed in a 96 well flat-bottomed microtiter cell culture plate with a diluted colony concentration of 1 × 10^6^ cfu/mL, and incubated at 37 °C for 16–20 h in a shaking incubator at different rpm, on the basis of different bacteria. After, plates were washed twice by PBS and incubated with 100 μL of Dermaseptin-PH dissolved in relevant culture medium, in a range of concentrations from 1 to 512 μM. Sixteen to twenty hours after exposure, plates were washed twice by PBS, and fixed with methanol for 10 min. Both MBIC and MBEC plates were stained with 150 μL 0.1% (*g*/*v*) crystal violet solutions for 30 min, and washed by PBS until no apparent stains were observed. Evaporating crystal violet stains were dissolved by 100 μL 30% (*v*/*v*) acetic acid, and the absorbance values were measured at wavelength 595 nm using a Synergy HT plate reader (Biotech, Minneapolis, MN, USA). The minimum concentration that inhibits the formation of biofilm is defined when compared to the negative control group as MBIC i.e., ≥90% (for MBIC_90_) and MBIC i.e., ≥50% (for MBIC_50_). The minimum concentration that eradicates the biofilm is defined when compared to the negative control group as MBEC i.e., ≥90% (for MBEC_90_) and MBEC i.e., ≥50% (for MBEC_50_) [41].

### 4.8. Bacteria Cell Membrane Permeability Assay of Dermaseptin-PH

Bacteria were inoculated in TSB (Sigma-Aldrich, St. Louis, MO, USA) at 37 °C for 16 to 18 h, and then subcultured until reaching the logarithmic growth phase, by measuring OD of the cultures at wavelength 550 nm. Then, bacterial cells were collected, centrifuged at 1000× *g* for 10 min at 4 °C, where after, cells were washed twice with 5% TSB in 0.85% NaCl solution. The pellet was resuspended with 5% TSB in 0.85% NaCl solution to obtain a cell concentration of 1 × 10^8^ cfu/mL by measuring optical density (OD) at wavelength 590 nm. The cells were incubated with Dermaseptin-PH in a range of concentrations based on the MIC results in a 96 well black plate at 37 °C. Two hours after exposure, the cells were stained with SYTOX^TM^ green nucleic acid stain (Life technologies) dissolved in 5% TSB in 0.85% NaCl to a final concentration of 5 μM, and incubated for 5min in the dark at 37 °C. The fluorescent intensity was measured with a Synergy HT plate reader (Biotech, Minneapolis, MN, USA) by an excitation and emission wavelength of 485 and 528 nm, respectively. Bacteria cells treated with 70% (*v*/*v*) isopropanol for 1 h were used as positive control.

### 4.9. Haemolysis Assay

The defibrinated horse blood (TCS Biosciences Ltd., Botolph Clayton, UK) was collected, centrifuged at 1000× *g* for 5 min, where after, cells were washed with PBS until the supernatant was clean. The cells were resuspended with PBS to obtain a cell concentration of 4% (*v*/*v*). The pellet was incubated with Dermaseptin-PH in a range of final concentration from 1 to 512 μM at 37 °C. Two hours after exposure, supernatant was collected, centrifuged at 1000× *g* for 5 min, and placed into a 96 well plate. The absorbance was measured at wavelength 570 nm using a Synergy HT plate reader (Biotech, Minneapolis, MN, USA). Cells treated with 1% (*v*/*v*) Triton-X 100 for 2 h were used as positive control.

### 4.10. MTT Cell Proliferation Assay

MCF-7 (ATCC-HTB-22), MDA-MB-435S (ATCC-HTB-129), and U251MG (ECACC-09063001) were cultured in DMEM (Invitrogen, Paisley, UK) supplemented with 10% (*v*/*v*) fetal bovine serum (FBS) (Sigma-Aldrich, St. Louis, MO, USA) and 1% (*v*/*v*) penicillin–streptomycin (Invitrogen, Paisley, UK). PC-3 (ATCC-CRL-1435) and H157 (ATCC-CRL-5802) were cultured in RPMI-1640 (Invitrogen, Paisley, UK) supplemented with 10% (*v*/*v*) FBS and 1% (*v*/*v*) penicillin–streptomycin. HMEC-1 (ATCC-CRL-3243) was cultured in MCDB131 (Gibco, Paisley, UK) supplemented with 10% (*v*/*v*) FBS, 1% (*v*/*v*) penicillin–streptomycin, 10 ng/mL epidermal growth factor (EGF), 10 mM l-glutamine, and 1 μg/mL hydrocortisone. Cells were grown in a 96 well plate (5000 cells/well/100 μL) for 24 h, and treated with relevant serum-free medium for 12 h. After, cells were treated with Dermaseptin-PH dissolved in relevant serum-free medium in a range of concentrations, from 10^−9^ to 10^−4^ M. Twenty-four hours after exposure, the cells were incubated with MTT (3-(4,5-Dimethylthiazol-2-yl)-2,5-Diphehyltetrazolium Bromide) dissolved in PBS to a final concentration of 0.5 mg/mL at 37 °C for 4 to 6 h. After, medium in each well was removed and 100 μL dimethyl sulfoxide (DMSO) was added. Cell viability was measured at wavelength 570 nm using a Synergy HT plate reader (Biotech, Minneapolis, MN, USA).

### 4.11. Cancer Cell Membrane Permeability Assay of Dermaseptin-PH

The permeability of Dermaseptin-PH on MCF-7 cell was determined using SYTOX^TM^ green nucleic acid stain (Life technologies). MCF-7 cells were grown in a sterile black 96 well plate (5000 cells/well/100 μL) for 24 h, and treated with relevant serum-free medium for 12 h. After, cells were incubated with SYTOX^TM^ green stain (1 μM) for 15 min and subsequently were treated with various concentrations of Dermaseptin-PH with final concentrations range from 10^−6^ to 10^−4^ M. The fluorescence was measured every 15 min for 75min with a Synergy HT plate reader (Biotech, Minneapolis, MN, USA) by an excitation and emission wavelength of 485 and 528 nm, respectively. Cells treated with 0.5% Triton X-100 were used as positive control.

## Figures and Tables

**Table 1:** Minimum inhibitory concentrations (MICs) and minimum bactericidal concentrations (MBCs) of Dermaseptin-PH, ampicillin (Amp) and norfloxacin (Nor) against tested microbes.

Strains	MIC (μM)			MBC (μM)		
	Dermaseptin-PH	Amp	Nor	Dermaseptin-PH	Amp	Nor
***Escherichia coli***	16	36.6	0.6	16	36.6	0.6
***Pseudomonas aeruginosa***	64	-	2.5	>512	-	5.2
***Staphylococcus aureus***	32	0.3	1.3	64	0.3	2.5
**MRSA**	>512	-	2.5	>512	-	5.2
***Enterococcus faecalis***	32	4.8	5.2	64	4.8	5.2
***Candida albicans***	16	-	1.3	32	-	2.5

**Table 2:** The antibiofilm activity of Dermaseptin-PH against *E. coli* and *S. aureus*.

Strains	MBIC_50_ (μM)	MBIC_90_ (μM)	MBEC_50_ (μM)	MBEC_90_ (μM)
*E. coli*	32	64	64	256
*S. aureus*	32	64	64	128

MBIC, minimum biofilm inhibitory concentration; MBEC, minimum biofilm eradication concentration; MBIC_50_, minimum biofilm inhibitory concentration required to inhibit the formation of 50% of biofilm; MBIC_90_, minimum biofilm inhibitory concentration required to inhibit the formation of 90% of biofilm; MBEC_50_, minimum biofilm eradication concentration required to eradicate 50% of the formed biofilm; MBEC_90_, minimum biofilm eradication concentration required to eradicate 90% of the formed biofilm.

## Abbreviations

The following abbreviations were used in this manuscript:

RACE	rapid amplification of cDNA ends
PCR	polymerase chain reaction
RP-HPLC	reverse-phase high-performance liquid chromatography
AMPs	antimicrobial peptides
HDPs	host-defence peptides
NCBI	National Centre for Biotechnology Information
BLAST	basic local alignment search tool
MALDI-TOF-MS	matrix-assisted laser desorption/ionization time-of-flight mass spectrometry
TFA	trifluoracetic acid
CHCA	*α*-cyano-4-hydroxycinnamic acid
TFE	2,2,2-trifluoroethanol
Fmoc	9-fluorenylmethyloxycarbonyl
